# Experimental study of solar air heater performance with evacuated tubes connected in series and involving nano-copper oxide/paraffin wax as thermal storage enhancer

**DOI:** 10.1007/s11356-022-22462-6

**Published:** 2022-08-16

**Authors:** Amr Elbrashy, Fawzy Aboutaleb, Magda El-Fakharany, Fadl Abdelmonem Essa

**Affiliations:** Mechanical Engineering Department, Faculty of Engineering, Kafrelsheikh University, Kafrelsheikh, 33516 Egypt

**Keywords:** Evacuated tube collector, Solar air heater, Nanoparticles, Paraffin wax, Thermal efficiency, Air flow rates

## Abstract

The investment of solar energy in life applications has become mandatory to maintain a clean environment and reduce the use of fossil fuels. This work aimed to improve the performance of solar air heater (SAH) by using evacuated tube solar collectors ETSC integrated with nano-enhancer phase change material (NE-PCM). To achieve this purpose, a system consisting of 5 linked collecting panels was designed, fabricated, and experimentally investigated. Each panel included a glass-evacuated tube with two concentric aluminum pipes installed inside. NE-PCM was placed between the inlet and outlet air paths inside the evacuated tube to enhance the heat transfer rate. The performance was investigated with and without NE-PCM at five mass flow rates (0.006, 0.008, 0.01, 0.03, and 0.05 kg/s). Experimental results revealed that the highest temperature was 116, 108, 102, 95, and 93 °C, respectively, for the above mass flow rates without adding NE-PCM. The outlet temperature was decreased by 6–15 °C when using NE-PCM. The SAH efficiency was increased by 29.62% compared to the system without NE-PCM at 0.05 kg/s. The maximum thermal efficiency for the system with NE-PCM was 62.66% at 0.05 kg/s, and the pressure drop was 6.79 kPa under the same conditions. As well known, the hot air is used for a variety of purposes including space heating, food processing, drying of fruit, vegetables, dairy, and solar cooking.

## Introduction

Day by day, the demand for energy is increasing rapidly over the world due to the depletion of fossil fuels and its effects on the environment. So, natural and renewable resources are promising solutions to satisfy our demands of energy. Natural and renewable resources are characterized by economic savings and environmental cleanliness. Solar energy is the most important natural resource because it has outstanding advantages such as vast availability, inexhaustibility, environmental friendliness, and extensive distribution. Also, solar energy provides various forms of energy such as benefit by transform into photovoltaic, thermal, and electric energy (in terms of thermal energy, solar collector is a well-known solar thermal energy conversion device that absorbs solar energy and converts it to a flowing fluid (water or air)). The most widely used solar collectors involve evacuated tube solar collectors (ETSC), flat-plate solar collectors (FPSC), and compound parabolic concentrators (CPCs) (Farhana et al. 2019). The solar air heater was widely used with FPSC because it is cheap and simple in construction. However, FPSC suffers from lower thermal efficiency due to low heat carrying capacity of air and less convective heat transfer coefficient between air and the absorber surface, which restricts the development of solar air heaters. Many modifications in the structure and design of FPSC systems are reported for performance development. To improve the thermal performance of air heaters in FPSC, Arunkumar et al. (2020) discussed all the different form disturbances used by researchers, including their shapes, orientations, and pitches. The addition of turbulators raises the temperature of the absorber duct outlet, and the pressure drop is also increased. As a result, the efficiency of turbulator is determined by the thermo-hydraulic enhancement factor values. However, due to its limits in higher temperature ranges (i.e., 70–95 °C) and poor performance, it has been suggested to be used for the evacuated tube and parabolic solar collector (Singh and Samsher. 2021). ETSC has gained considerable interest in solar energy field. It covers a wide range of working temperatures and provides better thermal efficiency. Besides, they have very small convective and radiative losses, all while being reasonably priced (Verma et al. 2020). Because of this, many researchers have been interested in applying the vacuum tube technique to solar air heaters (SAHs). Their efficiency has been studied, and some modifications have been inserted to improve heat transfer like baffles, helical, and concentrating reflective (Korres and Tzivanidis 2018; Pandey et al. 2021; Singh and Vardhan 2021; Veera Kumar et al. 2021). They found that the outlet temperatures reach to 100 °C. The average outlet temperature increases as the flow rate decreases, while the average thermal efficiency and pressure drop increase as the flow rate increases. In addition, the parabolic trough combined with ETSC produced more heat because it absorbs concentrated radiation. Moreover, the addition of different fins enhances heat transmission. But the length of the air path has a significant impact on heat transfer because it increases the heat exchange time, and this challenge leads to an increase in the number of evacuated tubes in order to extend the air path. Therefore, most of the published papers achieve the experiment between 15–30 vacuum tubes to reach the longest path. The connection in series between the evacuated tubes was the best solution to double the air path with the least number of evacuated tubes. However, this matter was investigated in a few papers (Pin-Yang Wang et al. 2014; Ping-Yang Wang et al. 2015). They reported that the outlet temperature reached 230 °C when using 30 units connected in series; each unit included a vacuum tube and CPC. In other research, the temperature dropped to 200 °C when using 10 units, indicating that controlling the number of connected tubes has a direct effect on the outlet temperature. Thermal energy storage is the sufficiently solution for the discontinuity and instability of solar energy. Therefore, many researchers are interested in studying the phase change materials (PCM) and using them with solar heaters in order to store heat for a longer time. In solar air heaters integrated with PCM, many researchers have experimented the effect of using acetamide (Mehla and Yadav 2018), paraffin (Abokersh et al. 2017; Algarni et al. 2020; Essa et al. 2018; Z. Wang et al. 2020), and other materials such as stearic acid, mannitol-graphite, and erythritol (Chopra et al. 2019; Li and Zhai 2017; Yongtai et al. 2019). All found that PCM increases the efficiency of thermal performance and thermal storage, where the heat lasts longer after the disappearance of sunlight. More details on evacuated tubes integrated with PCM were reviewed by Aramesh and Shabani (2020). Despite the common uses of paraffin wax as PCM, it is available in a wide range of melting points and latent heat capacities, low costs, nontoxicity, and physical and chemical stability; but their thermal conductivity is low which negatively affects the heat transfer rate. One of the solutions discussed to overcome the problem of heat transfer is the addition of nanomaterials, which have physical properties that increase the thermal conductivity (Qureshi et al. 2018). Numerous studies were conducted with adding nanoparticles at PCM with different ratios for enhancing the thermal conductivity of storage materials (Abdullah et al. 2020; Nižetić et al. 2020; Olfian et al. 2020). It was found that the range of thermal conductivity was increased by 20–100%. Nano-enhanced of phase change material (NE-PCM) integrated with ETSC has been discussed by many scholars. They pointed out that most of the NE-PCM applied in evacuated tube collectors were TiO_2_, CuO, Al_2_O_3_, CeO_2_, GNP, Cu, SWCNTs, and MWCNTs (Dsilva Winfred Rufuss et al. 2018; Ghaderian and Sidik 2017; A. Kumar et al. 2021; P. M. Kumar and Mylsamy 2020; Xiong et al. 2020). To increase the thermal performance of the system, it is critical to select the right material from among the available nanomaterials with the PCM. This emphasizes the need of evaluating the physical characteristics of the resultant composites, which will considerably influence the selection of acceptable materials as nanoparticles (GaneshKumar et al. 2022).

It is noticed from the literature that few studies have reported the use of ETSC in series connection. The investigations have depended on indirect contact surface between the ETSC as absorber and the U tube. So, in this paper, a new SAH was designed in series connection to increase the path of air and in direct contact between the air path and absorber to increase the heat transfer. Also, the SAH performance was enhanced using paraffin wax in combination with copper oxide nanoparticles (CuO) to boost its thermal conductivity. The modern distribution of NE-PCM was used to improve the charge and discharge processes. NE-PCM was placed between two tubes extending through the length of air path. The performance of the system was studied under different mass flow rates (0.006, 0.008, 0.01, 0.03, and 0.05 kg/s). This work is one of the important additions in the field of SAH integrated with NE-PCM and serves as a basis for future studies in the operation of SAH with ETSC connected in series.

## Experimental setup and methodology

This section is divided into three parts. The first one describes the materials and process of nano-PCM preparation. The second part describes the experimental setup, which consists of the solar air collector, instrumentation for measurement of various operating parameters, and the induction of nano-PCM into the system. The last part describes the methodology of experimental work.

### Materials

Paraffin wax is selected as PCM where it is reliable, chemically stable, stable for very long melting cycles, nontoxic, inexpensive, and available on the market. Therefore, it is chosen as a thermal storage material in solar collector systems. Table 1 displays the properties of the paraffin wax used in this study. However, paraffin wax is characterized by low thermal conductivity. So, to improve the thermophysical properties of paraffin wax, nanomaterials were added to it. CuO nanoparticles are more common in ETSC due to their high thermal conductivity related to the price and preparation method (Olfian et al. 2020). Table 2 shows the characteristics of the used CuO nanoparticles.

**Table 1.** Properties of paraffin wax used in this experimental

Property	Value
Melting temperature (°C)	59
Latent fusion heat (kJ/kg)	190
Solid state density (kg/m^3^)	920
Liquid state density (kg/m^3^)	790
Thermal conductivity (W/m K)	0.21
Specific heat (kJ/kg K)	2.3

**Table 2.** Characteristic of CuO nanoparticles

Property	Specification
Appearance	Black powder
Average size	45± 7 nm
Density	6300 kg/m^3^
Melting point	1250 °C
Boiling point	2000 °C
Thermal conductivity	33 W/m K

### NE-PCM preparation process

**Fig. 1.**
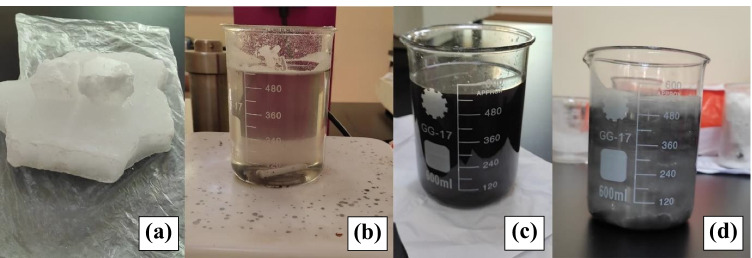
The stages of formation of the NE-PCM compound: **a** paraffin, **b** paraffin heated to 70 °C+ 0.2 % CuO nanoparticles, **c** mixture after ultrasonic, and **d** mixture at room temperature

The volume produced between the first and second aluminum tubes was calculated. Approximately 2.5 kg of paraffin wax was used and 5 grams of CuO nanoparticles was added as 0.2 % by weight fraction. A sample of paraffin wax (0.5 kg) was heated up to 70 °C and then 0.2 wt% of CuO nanoparticles was added to the paraffin to form NE-PCM nanocomposites by hot plate magnetic stirrer. The mixture was sonicated for 60 min at 30 kHz to ensure homogeneous mixing by ultrasonic vibrator type (TELSONIC ULTRASONICS CT-I2). NE-PCM was collected in containers and allowed to cool to an atmospheric temperature. Figure 1 depicts the stages of the formation of the NE-PCM compound.

### Experimental setup

**Fig. 2.**
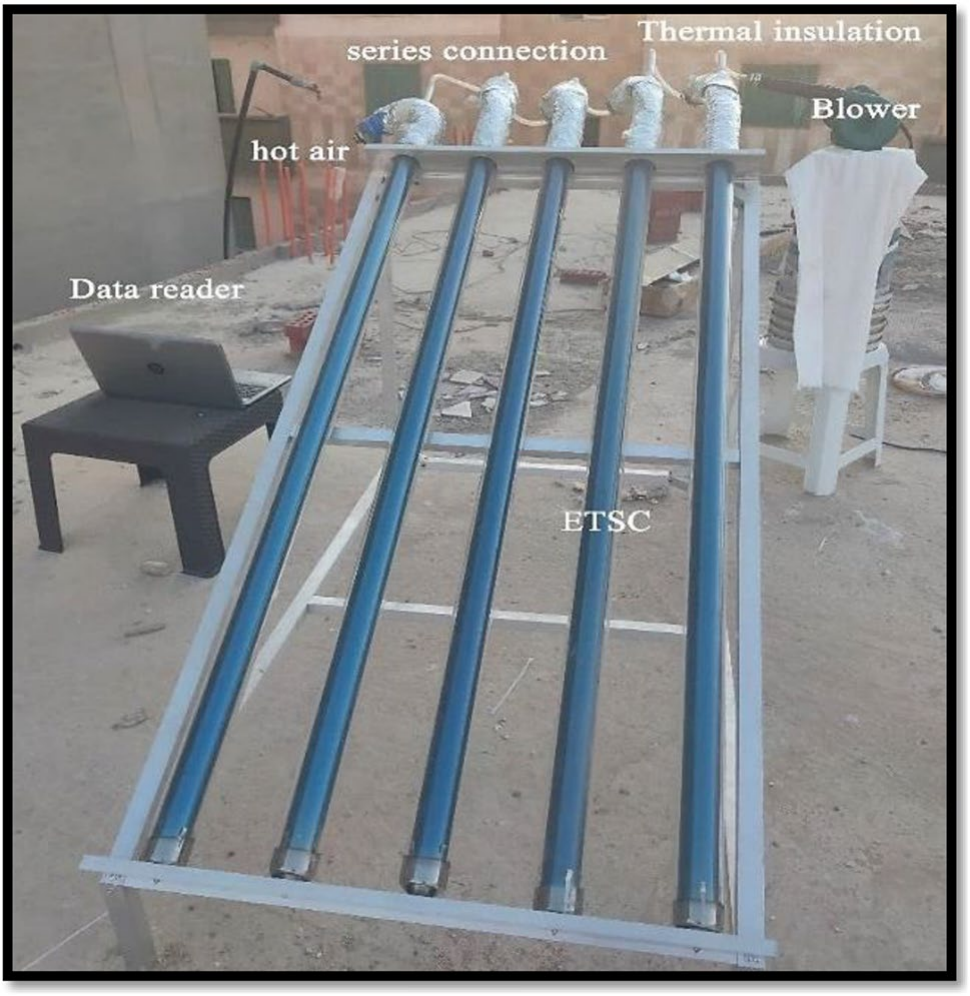
Photograph of the experimental work

To carry out the experimentations, the experimental setup consists of five units. Each unit contained an evacuated tube with 1800 mm in length and 58 and 47 mm for the outer and inner diameters, respectively. All tubes were inserted with two aluminum co-axial pipe of length 1700 mm. The first one was 16 mm and the second was 30 mm in diameter. Figure 2 shows a photographic view of the test device, which consists of the holder and five evacuated tubes containing two aluminum pipes connected in series. Aluminum tubes were welded together to form concentric pipe for inlet path of air and location for NE-PCM. The evacuated tubes were installed in a frame of metal inclined by angle of 30°, and at the top, each one was connected by flexible plastic hoses having dimensions of 12 mm and 65 mm to join the adjacent two units, and sealing tape was employed to prevent any leakage of air from the joints. The hoses were insulated using glass wool to prevent heat loss.

A centrifugal blower Trumax MX1040A has 650 W and volume flow rate is 2.8 m^3^/min as the maximum value has been connected to the collector for handling air. The incoming air from the blower was controlled by an adjusting valve to regulate air during experimentation.

**Fig. 3.**
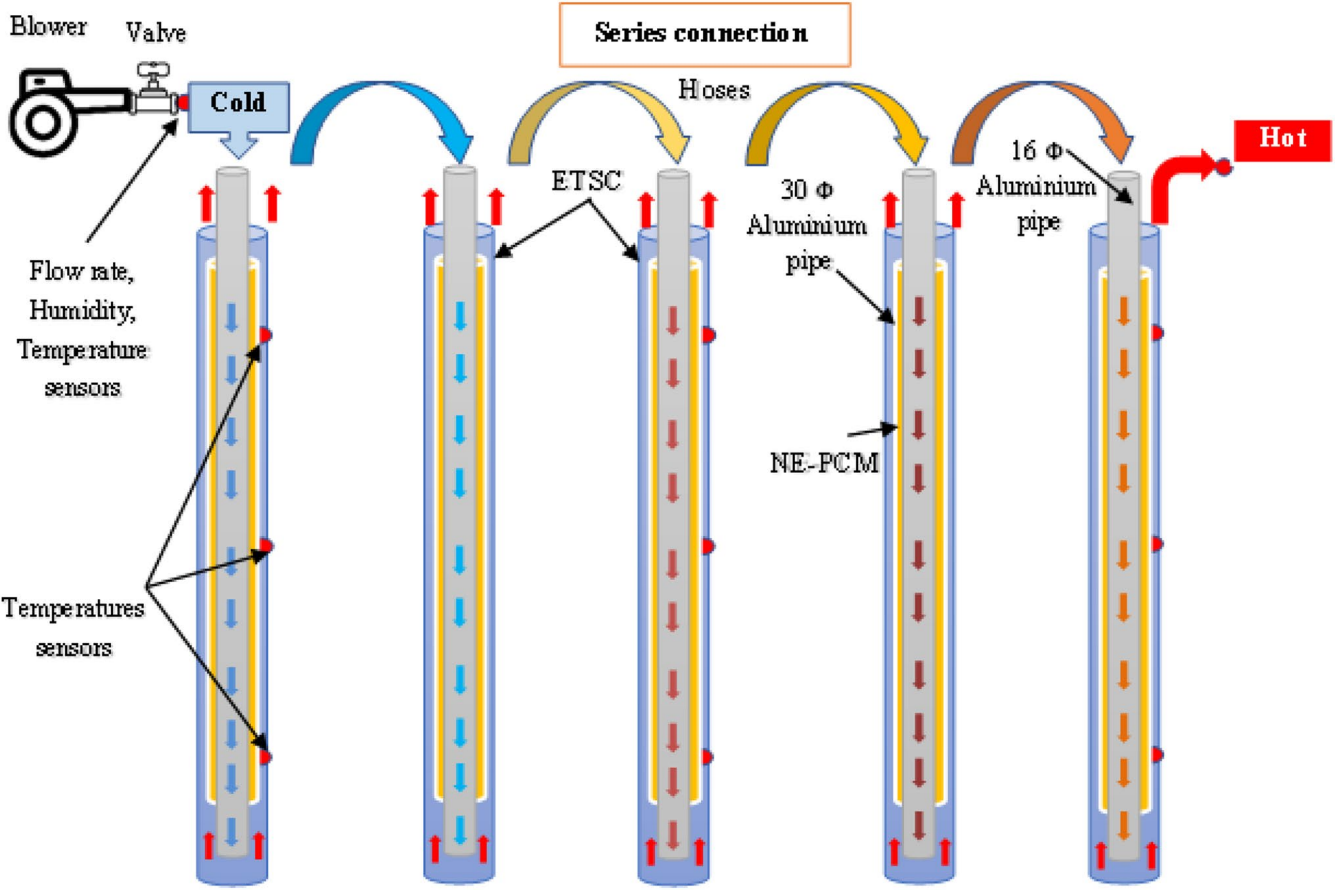
Schematic of sensors distribution within the system

Two temperature sensors were mounted at the inlet and outlet of the collector to measure air temperature flows. For the first, middle, and last units, three temperature sensors for each unit were mounted at the upper, middle, and lower locations respectively along the axial direction to measure the temperature distribution of NE-PCM. The photographic view and schematic of the experimental setup have been shown in Fig. 3. The detailed specifications of the system of the experimental setup are summarized in Table 3.

**Table 3:** Specifications of the test rig

Specification	Value
**Evacuated tube**	
Number of tubes	5
Length	1800 mm
Outer diameter (D_o_)	58 mm
Inner diameter (D_i_)	47 mm
Collector area	0.82 m^2^
Tube material	Borosilicate glass
Transmittance	0.90
Emissivity	0.045
Absorptivity	0.94
**Co-axial aluminum pipe**	
Outer diameter	16 mm
Inner diameter	12 mm
Length	1700 mm
**Aluminum pipe for NE-PCM**	
Outer diameter	30 mm
Inner diameter	27 mm
length	1200 mm
**Structure frame**	
Dimensions	147×85×75 mm
Inclined angle	30°
Material	Aluminum

### Experimental procedure

During the operation, air is pumped to pass through each collecting unit in the system and overcome the pressure drop. Controlled air enters through the first co-axial pipe, arrives at the bottom, and exits upwards from the evacuated tubes towards the top to enter the co-axial pipe of the second unit. Air is heated from coated inner tube of the evacuated tube by convective heat transfer, and heat is transmitted to the inlet air by thermal conduction from the axial tube. Therefore, the airflow inside the single pipe exceeds 3.5 m. Then, the total distance traveled by the airflow into the five units is approximately 18 m, during which the heat is gained. The temperature readings are observed at the inlet and at the outlet of the system, in addition, the temperature of the ambient air was recorded every 60 min.

### Measuring instruments

During the experiments, performance parameters are measured and recorded from 7:00 to 23:00. The temperatures are measured using LM35 temperature sensors of accuracy ± 0.5 °C connected to the Arduino board and then to the computer to read the data. The temperatures are measured at inlet and outlet of SAH for calculating temperature rise (ΔT). Also, nine temperature sensors were set in NE-PCM to record its temperature distribution. Moreover, the ambient temperature is measured and recorded. PYR-1307 pyranometer of ± 10 W/m^2^ accuracy is used to detect the intensity of solar radiation and the output from it is registered on the computer. In addition, the wind speed of the surrounding area across the collector surface is measured by an anemometer PS-2174 weather anemometer with ±0.17 m/s accuracy. Two DH22 sensors are used to measure the humidity at inlet and outlet of SAH. The pressure drop through SAH was measured using a BMP 280 sensor, and the values were checked by calculation from the next section. Table 4 shows the experimental sensors and accuracies.

**Table 4:** Specifications of measuring sensors

Sensor	Measurement	Unit	Accuracy
LM35	Temperature	°C	±0.5
DH22	Humidity	%	±2-5
BMP 280	Pressure	Pa	±12
Anemometer	Air velocity	m/s	± 0.17
Pyranometer	Solar radiation	W/m^2^	± 10

## Thermal performance analysis

### Thermal energy

The thermal efficiency is the ratio of useful thermal energy gained by the fluid to the heat absorbed by the solar collector area and intensity of radiation. The thermal efficiency of the system increases as the mass flow rates increase. The reason for this is that the reduction in pressure produces an increase in pumping power. The intensity of the solar radiation also increases the thermal performance, where the maximum value appeared at 13:00. To calculate the total thermal energy gain (*Q*_g_) from the system as a whole, the following can be used (Veera Kumar et al. 2021):

1 $${\mathrm{Q}}_{\mathrm{g}}=\dot{\mathrm{m}}\ {\mathrm{C}}_{\mathrm{p}}\ \left({\mathrm{T}}_{\mathrm{o}}-{\mathrm{T}}_{\mathrm{i}}\right)$$

where (*T*_o_-*T*_i_) is the difference between outlet and inlet temperatures (K). $\dot{\mathrm{m}}$ denotes the mass flow rate (kg/s), and *C*_P_ is the specific heat of air (J/kg K). The amount of actual thermal energy is absorbed by ETSC *Q*_a_ (W) which can be expressed by:

2 $${\mathrm{Q}}_{\mathrm{a}}=\mathrm{R}\ {\mathrm{A}}_{\mathrm{p}}\ \mathrm{N},$$

where *R* is the solar radiation (W/m^2^), *A*_p_ is the aperture area of collector, and *N* is number of evacuated tubes (Dhiman et al. 2011).

### Thermal efficiency

Thus, the effective efficiency of ETSC *η*_c_ can be calculated after the pressure loss is taken into account in the following:

3 $${\upeta}_{\mathrm{c}}=\frac{{\mathrm{Q}}_{\mathrm{g}}}{{\mathrm{Q}}_{\mathrm{a}}}$$

Because PCM has sensible and latent heat, the beneficial heat gain when engaging NE-PCM may be separated into three parts: when the PCM is in a solid state, heat gain is represented as:

4 $${\mathrm{Q}}_{\mathrm{g},\mathrm{pcm}}={\mathrm{m}}_{\mathrm{p}\mathrm{cm}}\ {\mathrm{C}}_{\mathrm{p},\mathrm{pcm}}\ {\left({\mathrm{T}}_{\mathrm{o}}-{\mathrm{T}}_{\mathrm{i}}\right)}_{\mathrm{p}\mathrm{cm}}$$

The thermal energy gains when the PCM undergoes a phase change process are estimated as follows:

5 $${\mathrm{Q}}_{\mathrm{g},\mathrm{pcm}}={\mathrm{m}}_{\mathrm{pcm}}\ {\mathrm{L}}_{\mathrm{pcm}}$$

where *L*_PCM_ is the latent heat for PCM in (J/kg). Finally, the efficiency of a collector equipped with pure or enhanced PCM can be assessed as follows (Algarni et al. 2020; Mehla and Yadav 2016):

6 $${\upeta}_{\mathrm{p}\mathrm{cm}}=\frac{\dot{\mathrm{m}}\ {\mathrm{C}}_{\mathrm{p}}\ \left({\mathrm{T}}_{\mathrm{o}}-{\mathrm{T}}_{\mathrm{i}}\right)}{\mathrm{R}\ {\mathrm{A}}_{\mathrm{p}}+\frac{\ {\mathrm{m}}_{\mathrm{p}\mathrm{cm}}\ {\mathrm{C}}_{\mathrm{p},\mathrm{pcm}}\ {\left({\mathrm{T}}_{\mathrm{o}}-{\mathrm{T}}_{\mathrm{i}}\right)}_{\mathrm{p}\mathrm{cm}}}{\uptau}+\frac{\ {\mathrm{m}}_{\mathrm{p}\mathrm{cm}}\ {\mathrm{L}}_{\mathrm{p}\mathrm{cm}}}{\uptau}}$$

### Pressure drop

The pressure drop can be calculated by taking the diameter *D* and the length *L* into account, and may be computed by the Darcy equation as follows (Bagherzadeh et al. 2019):

7 $$\Delta \mathrm{P}=\mathrm{f}\ \left(\frac{\mathrm{L}\uprho {\mathrm{V}}^2}{2\mathrm{D}}\right)$$

where *ρ*, *L*, *V*, and *f* denote the density, length of air path, flow air velocity, and the friction coefficient, respectively. Now, the density and dynamic viscosity of the air must be determined at the average temperature between the ambient and output temperature to simplify calculations.

The friction coefficient for smooth pipes may be determined as follows (Bagherzadeh et al. 2019):

8 $$\mathrm{f}=0.316\ \mathrm{R}{\mathrm{e}}^{-0.25}$$

where Re is the Reynolds number, which is defined as:

9 $$\operatorname{Re}=\frac{\uprho \mathrm{VD}}{\upmu}$$

The air velocity (*V*) was estimated at a flow air of 0.01 kg/s in the experiment. It can be calculated by (Pandey et al. 2021):

10 $$\mathrm{V}=\frac{\dot{\mathrm{m}}}{\uprho \left(\frac{\uppi}{4}\right){\mathrm{D}}^2}$$

### Uncertainty analysis

Instrumental error, environmental variables, measurement and observation errors, adequate experimental design, etc. have caused experimental uncertainty. In this investigation, several sources of uncertainty were addressed throughout the tests, such as temperature, humidity, flow velocity, intensity of radiation, pressure drop, and mass flow rate. The overall measurement of uncertainty and error analysis was carried out in the way reported by Singh and Vardhan (2021).

Thus, the total uncertainty of direct and indirect measurements can be calculated according to Equation (11) and Table 4.

11 $$U=\sqrt{{\left(\frac{\partial U}{\partial {x}_1}\delta {x}_1\right)}^2+{\left(\frac{\partial U}{\partial {x}_2}\delta {x}_2\right)}^2+\dots +{\left(\frac{\partial U}{\partial {x}_n}\delta {x}_n\right)}^2}$$

where ∂*x*_1_, ∂*x*_2_, ∂*x*_3_, …: ∂*x*_n_ are feasible uncertainty in measurements of *x*_1_, *x*_2_, *x*_3_, *x*_n_…. ∂U is known as absolute uncertainty, where ∂ xi denotes possible errors in measurements (x1= error in radiation, x2 = error in air velocity, x3= error in ambient temperature, and x4 = error in outlet temperature).

The uncertainty values found during experiments in terms of equipment performance were very small. All of the readings recorded during measurements in this study were within the permissible range. The uncertainty in measurements was used to assess the uncertainty in daily thermal efficiency. The highest value of uncertainty in daily thermal efficiency with and without storage material was found ± 3.28%.

## Result and discussion

The present experiments were done during the months of June, July, and August 2021 at Kafrelsheikh city (31.416667° N, 31.821389° E), Egypt. The results were registered from 7:00 to 23:00 to investigate the performance of SAH by increasing the length of the air pathway and enhancing phase change materials with nanoparticles.

The effects of weather conditions, mass flow rate, solar radiation, and addition of NE-PCM on the thermal performance of SAH, pressure drop, and outlet temperature have been studied in the subsequent sections. The results were compiled daily, every 1 h under different climatic conditions such as ambient temperature 30–40 °C, humidity 50–70%, wind speed 1–3 m/s, and solar radiation intensity 850–1120 W/m^2^. The ambient temperature increases directly with the solar radiation, as discussed in Abi Mathew and Thangavel (2021). In the month of July, the ambient temperature was recorded between 36 and 40 °C. Table 5 lists the average values during the months of the experiment for solar radiation, ambient temperature, wind speed, and humidity. Solar flux starts from 7:00 to 17:00; this means the ability to extract solar energy about 10 h a day. The results of experimental measurements have been depicted and discussed in detail below:

**Table 5:** Average value of weather parameter at different months

Parameter	June	July	August
Solar radiation	1034 W/m^2^	1070 W/m^2^	992 W/m^2^
Ambient temperature	34 °C	36 °C	33 °C
Wind speed	1.5 m/s	1.8 m/s	2.5 m/s
Humidity	63 %	68%	52 %

### Effect of weather conditions on outlet temperature

**Fig. 4.**
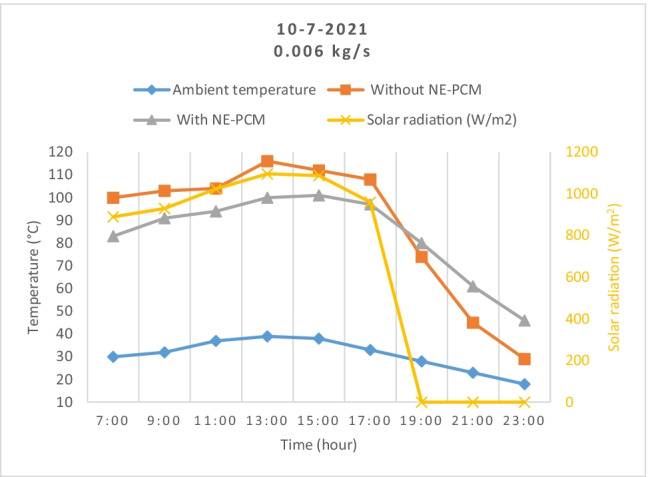
Outlet temperatures variation during the day as solar radiation changes

**Fig. 5:**
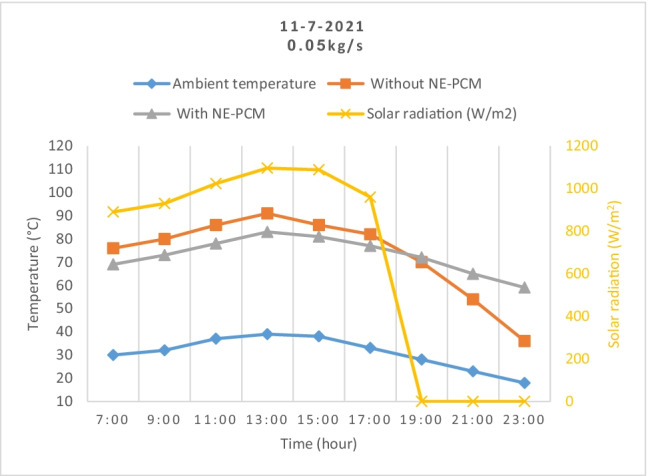
Outlet temperatures variation with and without NE-PCM as ambient temperature changes

The solar radiation, ambient temperature, and temperatures of the outlet air from the SAH with and without storage materials variations were measured with time at air mass flow rates of 0.006 and 0.05 kg/s as shown in Fig. 4 and Fig. 5, respectively.

The results show that the temperature increases from 7:00 until it reaches its maximum at 13:00. Then, it remains very close until 15:00. After that, it decreases to the end of day. It has the same trend of the solar radiation throughout the daytime. From results, it can be noted that the outlet air temperature without NE-PCM was greater than the outlet air temperature with NE-PCM during the charge period. The maximum outlet air temperature without NE-PCM was 93 and 86 °C while with NE-PCM was 100 and 116 °C at flow air of 0.05 and 0.006 kg/s, respectively. This is because the aluminum pipes and NE-PCM absorbed some heat during the flow of air inside the system for charging. During the discharge period after sunset, the heat will transfer from NE-PCM to the air, causing a decrease in the temperature of the NE-PCM. So, the outlet air temperature without NE-PCM drops rapidly compared to the outlet air temperature with the presence of NE-PCM.

The average ambient temperature was 28.5°C, while the highest value was 39°C at 13:00. The highest value of temperature rise has been recorded between the inlet and outlet air as 54 and 77 °C at 0.05 and 0.006 kg/s, respectively.

### Effect of mass flow rate on outlet temperature

**Fig. 6:**
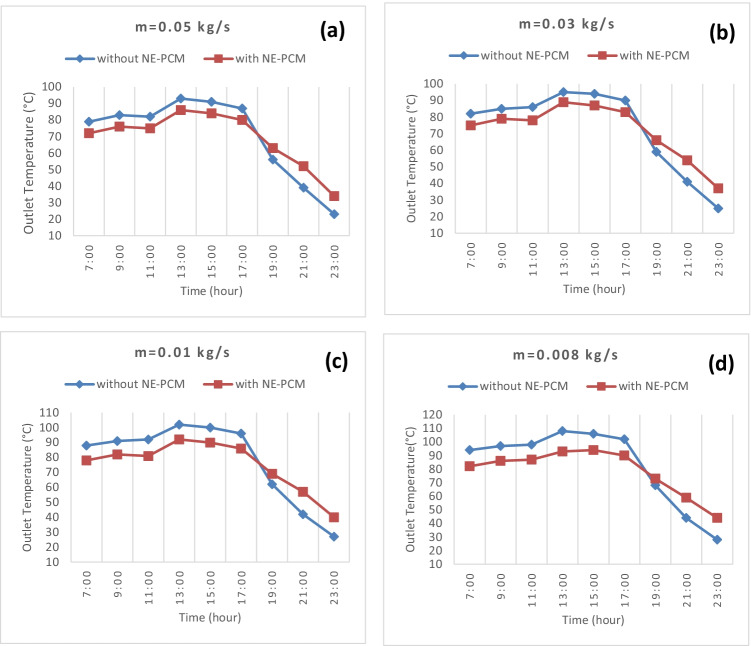
Effect of mass flow rate on outlet temperature through experimental time at different flow rates

Figure 6 indicates the four plots of outlet air temperature with and without NE-PCM at four studied air flow rates with time. The results show the outlet air temperature is inversely proportional to the mass flow rate, where the temperature at the exit increases as the flow rate of the mass decreases. It is clear from the figures that the high potential for high outlet air temperatures is at a low flow rate. Also, for all air flow rates, the outlet temperature without NE-PCM was higher than with NE-PCM until sunset, then started falling. For the four mass flow rates 0.008, 0.01, 0.03, and 0.05 kg/s, the highest air outlet temperature was achieved at 13:00 where the values were 94, 92, 89, and 87 °C, respectively, in case without NE-PCM and 108, 102, 95, and 93 °C, respectively, in the case with NE-PCM.

### Useful energy gain

**Fig. 7:**
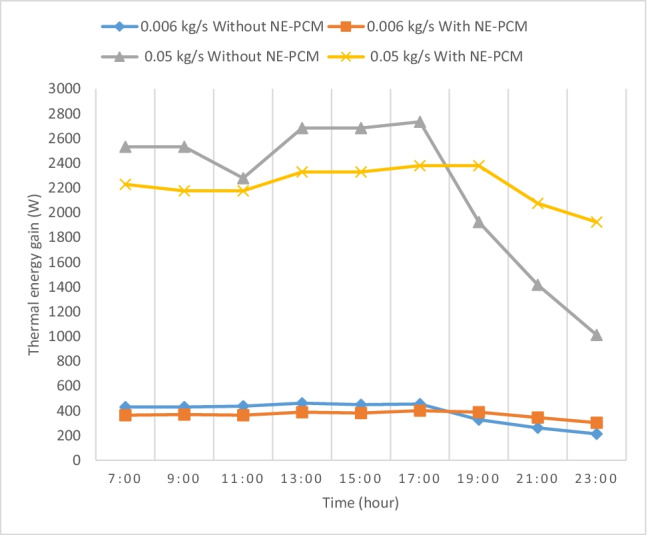
Variation of the useful energy gain with time at maximum and minimum flow rate

The variation of the useful energy gain extracted by the flowing air calculated from Eq. (1) for SAH with and without NE-PCM at the different air flow rates is illustrated in Fig. 7. It is noted that the trend of useful thermal energy was the same as that of solar radiation and outlet air temperature. It increases with time from 7:00 until it reaches its maximum at 13:00, then it begins to decrease with time because the useful energy gain depends on the outlet air temperature of SAH. It is observed that useful energy rises with increasing the air flow rates. The maximum increase of the useful energy for SAH with and without NE-PCM at 0.006 and 0.05 kg/s is about 911 and 91 W respectively, at 23:00.

### NE-PCM temperature during the charge and discharge process

**Fig. 8:**
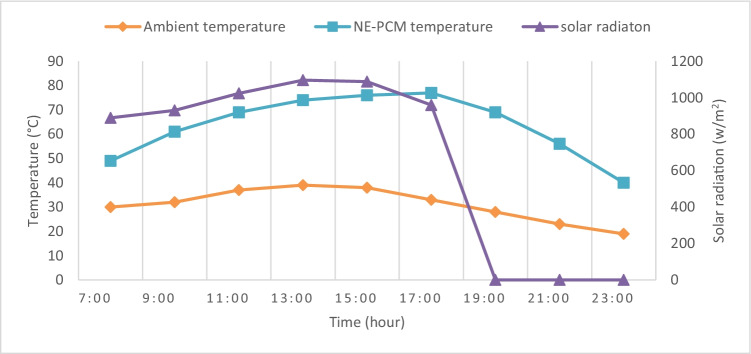
NE-PCM and ambient temperature during the experimental time

During the charging process, NE-PCM acquires the heat from the solar flux and stores it, so the NE-PCM begins to melt, and its temperature begins to rise. The average temperature variation of NE-PCM within the units was measured through a day and after sunset with time is observed as shown in Fig. 8. The NE-PCM temperature rises gradually in the period from morning until 17:00; when NE-PCM absorbed heat from hot air, during the discharge period, it drops due to the cold flowing air passing around it.

**Fig. 9:**
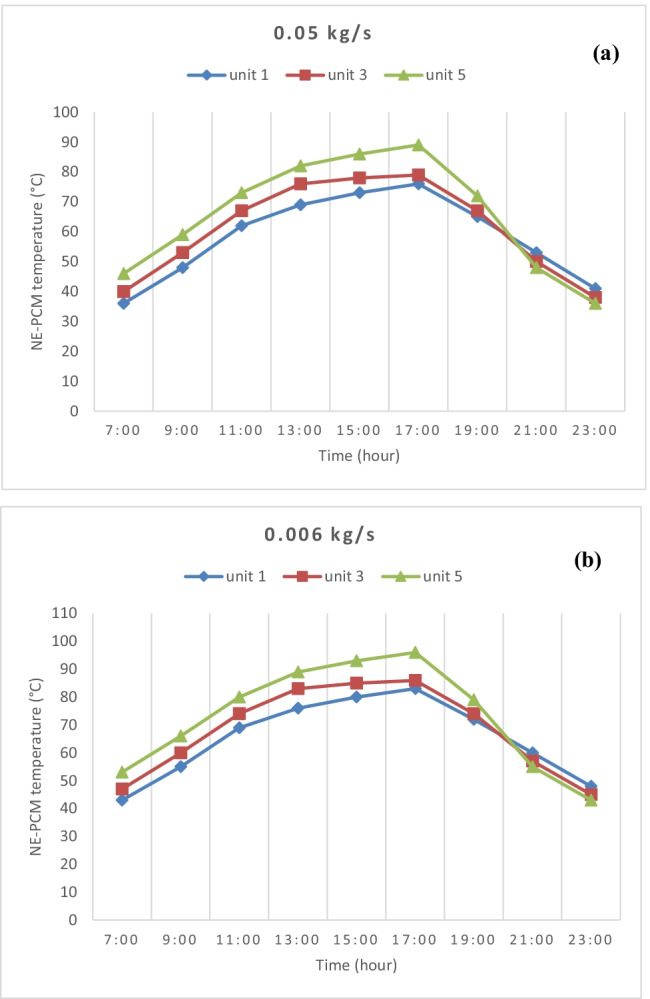
NE-PCM temperature for measuring units during the experimental time at **a** 0.05 kg/s and **b** 0.006 kg/s

The temperature sensors were placed within the NE-PCM of three units; the first, middle, and last one to measure the temperature change within them. The temperature of NE-PCM during operation in studied units with time at different flow rates is shown in Fig. 9a and b. During system operation, the air trajectory and flow rate have a major impact on the temperature of NE-PCM. The lower air flow rate, the greater heat transmission because of the air friction with the solar flux absorbed from the collector. As can be noted that before 17:00, the NE-PCM temperature of each measuring unit increases and falls after that according to solar radiation intensity. The temperature increases from first unit to the last due to the series connection of SAH, where the air flow path starts from the first unit to the last unit. The highest temperature was 76, 79, and 89 °C for the first unit, third, and fifth respectively at 0.05 kg/s. NE-PCM temperature for each measuring unit decreases with increasing the air flow rate; it ranged between 2 and 7 °C, for the first unit, and 2 and 9 °C for the last at 0.05 kg/s and 0.006 kg/s, respectively.

**Fig. 10:**
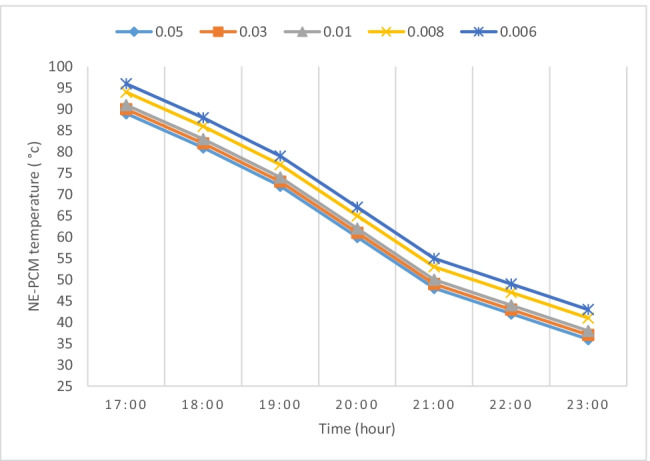
NE-PCM temperature in the last unit during discharge at different flow rates

Figure 10 shows the temperature of NE-PCM within the fifth unit during the discharge period at various air flow rates (0.006, 0.008, 0.01, 0.03, and 0.05 kg/s). NE-PCM temperature rapidly decreases with time for all air flow rates. However, NE-PCM temperature with 0.05 kg/s is lower than 0.006 kg/s because the heat stored in NE-PCM is quickly removed. At the beginning of the discharging process, NE-PCM temperature is high approximately 96, 94, 91, 90, and 89 °C at air flow rates (0.006, 0.008, 0.01, 0.03, and 0.05 kg/s) respectively. In the last stage of the discharging process, the NE-PCM temperature is close to the ambient temperature because the addition of nano on PCM improves the thermal conductivity through charge and discharge. It is about 43, 41, 38, 37, and 36 °C at air flow rates 0.006, 0.008, 0.01, 0.03, and 0.05 kg/s, respectively.

### Distribution of temperatures in characteristic points of the system

**Fig. 11:**
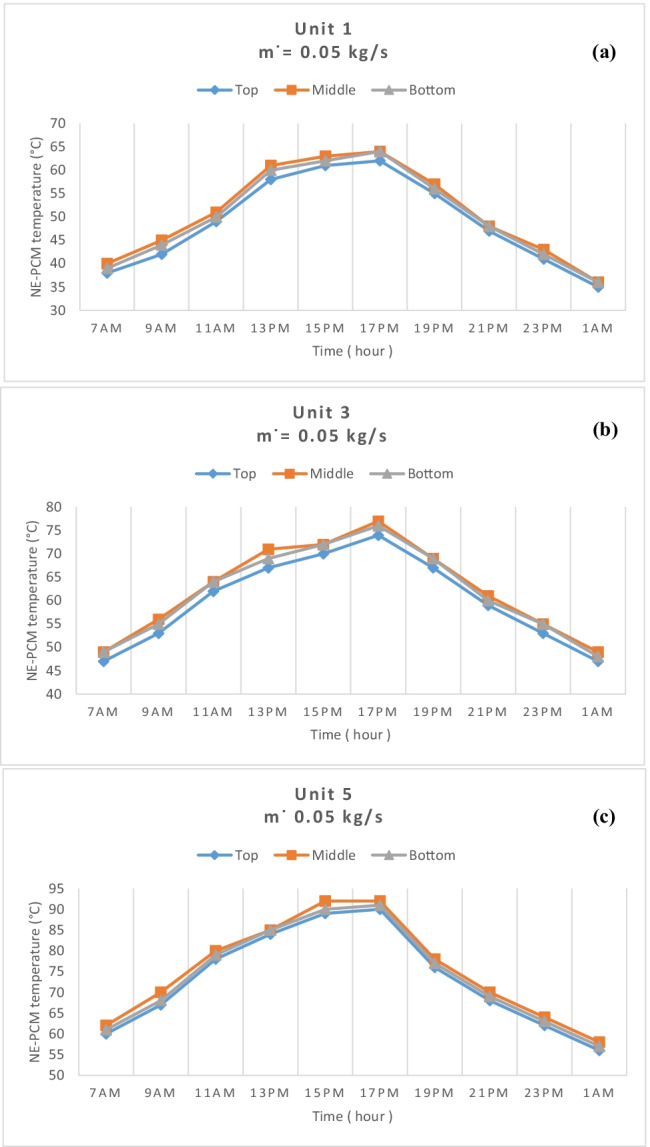
Temperature distributions in **a** the first, **b** third, and **c** fifth unit at 0.05 kg/s

**Fig. 12:**
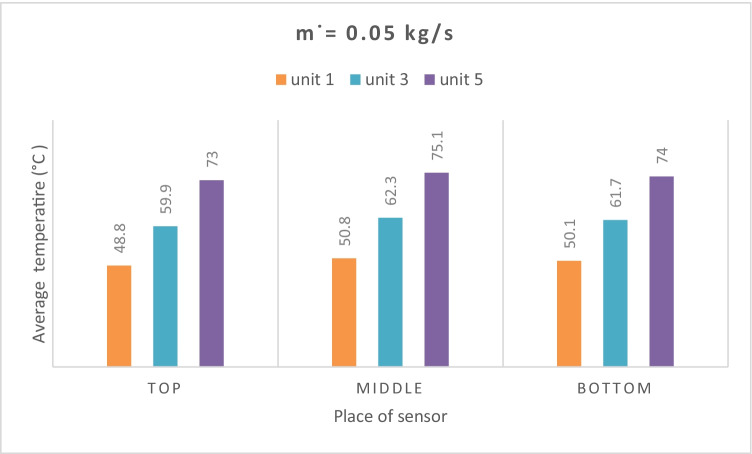
Average temperature distribution in three units through experimental time at 0.05 kg/s

The temperature distribution of each measuring unit is measured at three points: at the top, middle, and bottom. The system is designed for air flow paths rounded by the NE-PCM to improve the charge and discharge process where the low air temperature is inside and high from the outside. Figure 11a, b, and c shows the measured temperature distribution of NE-PCM for each measuring unit at 0.05 kg/s air flow rate. It can be seen from Fig. 11 that the difference in temperature between the top, middle, and bottom points is slightly low. It has the same trend for all measured units. So, it can be said that one sensor at middle of each measured units is enough to measure the NE-PCM temperature. Figure 12 shows the average temperature distribution for measuring units at different points with various air flow rates. The average temperature distribution through the unit is very small, while the difference between the measurement units is observed due to the series connection of the system. The average temperature values for NE-PCM were recorded at the top of the first, third, and fifth tubes 48.8, 59.9, and 73 °C, respectively, while for the middle tube, it was recorded as 50.8, 62.3, and 75.1 °C respectively. Furthermore, in the bottom of the unit, the average temperatures are recorded as 50.1, 61.7, and 74 °C at the first, second, and third units, respectively.

The average temperature values for NE-PCM were recorded at the top of the first, third, and fifth tubes as 48.8, 59.9, and 73 °C, respectively, while for the middle tube, it was recorded as 50.8, 62.3, and 75.1°C respectively. Furthermore, in the bottom of the unit, the average temperatures were recorded as 50.1, 61.7, and 74 °C at the first, second, and third units, respectively.

### Thermal performance and storage efficiency

**Fig. 13:**
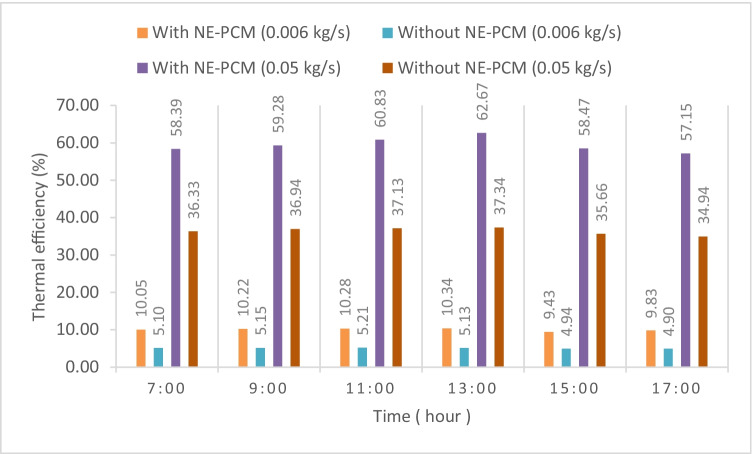
Thermal efficiency with and without NE-PCM at 0.05–0.006 kg/s

**Fig. 14:**
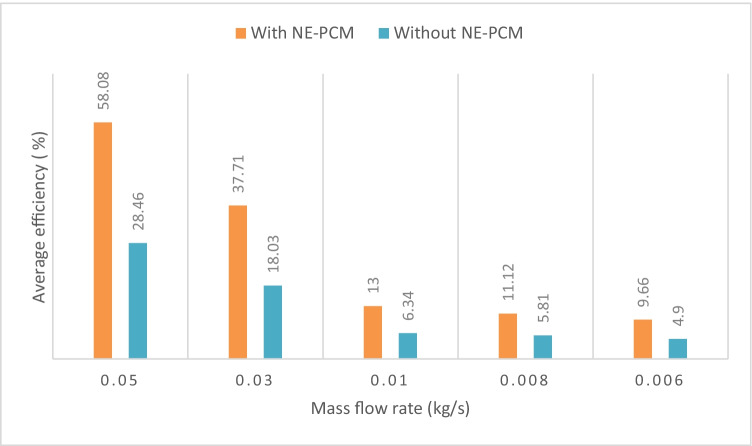
Average daily efficiency at measured air flow rates with and without NE-PCM

One of the important indicators used to evaluate the performance of the energy conversion process is the thermal efficiency of the collector. The thermal efficiency variation with and without NE-PCM is calculated based on Eqs. (3) and (6) for the measured air flow rates. The calculated thermal efficiency with and without NE-PCM at 0.006 and 0.05 kg/s air flow rates is presented in Fig. 13. It can be noted that the thermal efficiency variations of SAH with and without NE-PCM are small in the daytime. It slightly increases until 13:00. Then, it decreases for the rest of the daytime. It largely increases by using NE-PCM. It was 62.66% and 37.34%, for the mass flow rate of 0.05 kg/s with and without NE-PCM, respectively. Also, the efficiency increases with an increase in the air flow rate; it is obvious that it depends strongly on the air flow rate. The difference rise in thermal efficiency between mass flow rates of 0.006 and 0.05 kg/s was 32.21 % and 52.32 % respectively due to increases in heat gain. The average daily efficiency as a function of measured air flow rate with and without NE-PCM is demonstrated in Fig. 14. The average efficiency with NE-PCM is greater than average efficiency without NE-PCM in all air flow rates. The average efficiency of the SAH without NE-PCM is about 4.9%, 5.81%, 6.34%, 18.03%, and 28.46% for the air flow rates of 0.006 kg/s, 0.008 kg/s, 0.01 kg/s, 0.03 kg/s, and 0.05 kg/s, respectively, with an increase of about 4.76 %, 5.31 %, 6.66 %, 19.68 %, and 29.62 % respectively compared to SAH with NE-PCM.

## Conclusion

In this study, the thermal performance of solar air heaters with ETSC linked in series to increase the passage of air inside the system units is evaluated. To achieve higher performance, SAH is integrated with copper oxide nanoparticles enhanced paraffin wax to improve the thermophysical properties. SAH performance without NE-PCM is compared with SAH performance with NE-PCM. Based on the findings, the following conclusions are arrived:

- Connecting five units in a series showed a significant rise in temperature thanks to the doubling of the air path as well as direct contact, which increases the rate of heat transmission.
- The highest temperature recorded was 116, 108, 102, 95, and 93 °C at 13:00 for the mass flow rates of 0.006, 0.008, 0.01, 0.03, and 0.05 kg/s respectively in July without adding NE-PCM. Increases or decreases in temperature depend on the intensity of solar radiation and the change in the surrounding conditions.
- The proposed SAH device achieved maximum thermal efficiencies of 62.66% and 37.34% with and without NE-PCM respectively at 0.05 kg/s, and the pressure drop was 6.79 kPa.
- When NE-PCM was added to the system, a decrease in the output temperature from 6 to 15 °C was observed for air flow rates of 0.006 kg/s, 0.008 kg/s, 0.01 kg/s, 0.03 kg/s, and 0.05 kg/s. In contrast, thermal efficiency was increased by 4.76 %, 5.31 %, 6.66 %, 19.68 %, and 29.62 % respectively compared to SAH without NE-PCM.
- The maximum useful energy gained for SAH with and without NE-PCM at 0.006 kg/s, and 0.05 kg/s was about 2735 W, 2380 W, 426 W, and 389 W, respectively.
- Average NE-PCM temperatures in the first, third, and fifth units are 50, 62, and 75 °C respectively at 0.05 kg/s. It is worth mentioning that the temperature distributed down, middle, and top of the unit is almost the same.
- The tested air heater is used in temperature applications ranging from 80 to 120 °C, such as the heating space and conditioning in residential areas; drying of fruit, vegetables, and dairy products; taking moisture and operational time into account; and solar cooking.

## Data Availability

The datasets used and/or analyzed during the current study are available from the corresponding author upon request.

## Nomenclature

*A*_p_: Aperture area solar collector (m^2^)
*C*_p_: Specific heat of air (J/kg K)
*D*: Diameter of tube (m)
*m*˙: Mass flow rate of air (kg/s)
*m*_pcm_: Mass of the PCM (kg)
*L*_PCM_: Latent heat for PCM in (J/kg)
*N*: Total number of evacuated tubes
*Q*_g_: Heat gain (W)
*Q*_g,pcm_: Thermal energy gains when the PCM undergoes a phase change process (J)
*Q*_a_: Actual heat absorbed to collector (W)
*R*: Solar radiation (W/m^2^)
Ta: Ambient temperature (°C)
*T*_o_-*T*_i_: Change in temperature (K)
*V*: Velocity of air (m/s)
Re: Reynolds number
*f*: Friction factor of the smooth duct

## Abbreviations

CPC: Compound parabolic collector
FPSC: Flat-plate solar collector
ETSC: Evacuated tube solar collector
PCM: Phase change material
NE-PCM: Nano-enhanced PCM
SAH: Solar air heater

## Greek symbols

*ρ*: Density (kg/m^3^)
*τ*: Time
*μ*: Dynamic viscosity of air (Pa.s)
ΔP: Pressure drop (Pa)
*η*_c_: Efficiency of the collector
*η*_pcm_: Efficiency of the collector equipped with PCM
